# Structural and electronic properties of two-dimensional stanene and graphene heterostructure

**DOI:** 10.1186/s11671-016-1731-z

**Published:** 2016-11-25

**Authors:** Liyuan Wu, Pengfei Lu, Jingyun Bi, Chuanghua Yang, Yuxin Song, Pengfei Guan, Shumin Wang

**Affiliations:** 1 State Key Laboratory of Information Photonics and Optical Communications, Ministry of Education, Beijing University of Posts and Telecommunications, P.O. Box 72, Beijing, 100876 China; 2 School of Physics and Telecommunication Engineering, Shanxi University of Technology (SNUT), Hanzhong, 723001 Shaanxi China; 3 Beijing Computational Science Research Center, Beijing, 100084 China; 4 State Key Laboratory of Functional Materials for Informatics, Shanghai Institute of Microsystem and Information Technology, Chinese Academy of Sciences, Shanghai, 200050 China; 5 Photonics Laboratory, Department of Microtechnology and Nanoscience, Chalmers University of Technology, 41296 Gothenburg, Sweden

**Keywords:** First-principles, Stanene, Graphene, Heterostructure, Structural properties

## Abstract

Structural and electronic properties of two-dimensional stanene and graphene heterostructure (Sn/G) are studied by using first-principles calculations. Various supercell models are constructed in order to reduce the strain induced by the lattice mismatch. The results show that stanene interacts overall weakly with graphene via van der Waals (vdW) interactions. Multiple phases of different crystalline orientation of stanene and graphene could coexist at room temperature. Moreover, interlayer interactions in stanene and graphene heterostructure can induce tunable band gaps at stanene’s Dirac point, and weak p-type and n-type doping of stanene and graphene, respectively, generating a small amount of electron transfer from stanene to graphene. Interestingly, for model $\mathrm{S}\mathrm{n}\left(\sqrt{7}\right)/\mathrm{G}(5)$ *,* there emerges a band gap about 34 meV overall the band structure, indicating it shows semiconductor feature.

## Background

Two-dimensional (2D) materials, such as graphene [1–6], silicene [7–13], germanene [14–16], hexagonal boron nitride (hBN) [17, 18], and transition metal dichalcogenides (TMDs, such as MoS_2_) [19, 20], have received considerable attention recently because of their outstanding properties and potential applications. These 2D layers can be integrated into a multilayer stack (vertical 2D heterostructure) and have been widely studied experimentally and theoretically, such as graphene/silicene (G/Si) [21, 22], graphene/hexagonal boron nitride (G/hBN) [23, 24], silicene/HBN [25], silicene/GaS [26, 27], TMDCs/graphene [28, 29], stacked TMDCs [30, 31], phosphorene/MoS2 [32], and phosphorene/graphene [33]. The resulting artificial 2D heterostructures provide access to new properties and applications far beyond their simplex components.

Most recently, a new 2D material, stanene (the form of 2D stannum), firstly proposed by Liu et al. [34], has been mentioned as a host material for topological insulator (TI), which are new states of quantum matter with an insulating bandgap in the bulk while conducting states at the edges and protected by time reversal symmetry [35–40]. For instance, stanene and its derivatives could support a large-gap 2D quantum spin Hall (QSH) state and thus enable the dissipation less electric conduction at room temperature. Moreover, stanene could also provide enhanced thermoelectricity [41], topological superconductivity [42], and the near-room-temperature quantum anomalous Hall (QAH) effect [43]. Zhu et al. [41] have reported the successful fabrication of 2D stanene with metallic features on the Bi_2_Te_3_ (111) substrate by molecular beam epitaxy (MBE). Xu et al. [44] found that varying substrate conditions AB(111), where A = Pb, Sr, Ba and B = Se, Te, considerably tunes electronic properties of stanene, and the supported stanene gives either trivial or QSH states, with significant Rashba splitting induced by inversion asymmetry.

Technically, it is possible to fabricate a heterostructure of stanene on a suitable substrate, in order to form honeycomb-like bilayer atomic structure. Stanene has a hexagonal lattice, as well as the requirement of lattice status of the substrate. The lattice mismatch between the substrate and the stanene should be small, and it should be energetically favorable to stanene to grow in a quasi-two-dimensional growth mode. As one of the popular 2D materials, we propose a question whether stanene can grow on a graphene substrate or stanene/graphene (Sn/G) can form a 2D heterostructure with promising structural and electronic properties.

In this work, we design a new 2D stanene/graphene heterostructure and study its geometric and electronic properties by using first-principles calculations. The results show that stanene interacts overall weakly with graphene via vdW interactions. Therefore, their intrinsic electronic properties can be preserved in stanene/graphene heterostructure. Moreover, interlayer interactions in stanene/graphene heterostructure can induce tunable band gaps at stanene’s Dirac point, and weak p-type and n-type doping of stanene and graphene, respectively. Our paper is organized as follows. In the “Methods” section, we describe the details of computational methods. The results and discussions are presented in the “Results and Discussion” section. Finally, a brief summary is summarized in the “Conclusions” section.

## Methods

Our theoretical calculations are performed in the framework of density functional theory (DFT) [45] as implemented in the Vienna ab initio simulation package (VASP) [46]. Valence wave functions are treated by the projector augmented wave (PAW) [47, 48] method that uses pseudopotential operators but keeps the full all-electron wave functions. The interlayer interaction is checked by various exchange-correlation energy functionals, including the local density approximation (LDA) [49], the Perdew–Burke–Ernzerhof (PBE) [50] generalized gradient approximation (GGA), and the PBE with vdW corrections: the vdW-D2 functionals [51]. The plane-wave energy cutoff is set to be 400 eV. We have checked the convergence of k points, and a 5 × 5 × 1 k-sampling generated by the Monkhorst–Pack scheme [52] with Gamma centered for the Brillouin zone is adopted. The structural optimization is allowed to relaxed until the maximum force on each atom becomes at least less than 0.01 eV/Å and the maximum energy change between two steps is smaller than 10^−5^ eV. A vacuum layer of at least 20 Å is used.

## Results and Discussion

### Geometry and Energetics of Stanene/Graphene

For the monolayer graphene and free-standing low-buckled stanene, the lattice constants we obtained from LDA are 2.45 and 4.56 Å, respectively, which agree well with the reported values of 2.46 and 4.67 Å for graphene and stanene, respectively [53, 54]. Note that the lattice mismatch is as large as 7% even when a supercell consisting of 2 × 2 lateral periodicity of graphene and 1 × 1 stanene is employed. And the matched structure usually forms when the mismatch is small. An appropriate supercell in the bilayer system can be obtained by inducing relative rotations between the stanene and graphene substrates. For a 2D hexagonal lattice, it can be realized to get various lattice angles by longer lattice vectors from the primitive unit cell. For example, the angles corresponding to the lattice vectors for $\sqrt{3}\times \sqrt{3}$, $\sqrt{7}\times \sqrt{7}$, $\sqrt{13}\times \sqrt{13}$, $\sqrt{21}\times \sqrt{21}$, $\sqrt{31}\times \sqrt{31}$, $\sqrt{73}\times \sqrt{73}$, and $\sqrt{97}\times \sqrt{97}$ unit cells are 30°, 19.1°, 13.9°, 10.9°, 9.0°, 5.8°, and 15.3°, respectively.

The heterostructures along with their structural parameters are listed in Table 1. Taking the $3/\sqrt{31}$ for Sn/G as an example, it corresponds to a heterostructure consisting of 3 × 3 stanene unit cell and $\sqrt{31}\times \sqrt{31}$ graphene unit cell combined by a relative rotation of angle *α* equal to 9. This configuration will be represented as $\mathrm{S}\mathrm{n}(3)/\mathrm{G}\left(\sqrt{31}\right)$ below.

**Table 1:** Heterostructure configurations for the stanene/graphene bilayers (abbreviated as Sn/G)

Sn/G	*a* (Å)	*α* (°)	*θ* (°)	Δ (Å)	*L*_Sn_	*L*_G_	*L*_Sn/G_	Mismatch (%)	Strain (%)	*E*_b_ (meV)
$3/\sqrt{31}$	4.53	9	111.2	0.84	13.68	13.61	13.62	0.07	−0.4	−76
$2\sqrt{7}/\sqrt{97}$	4.55	3.8	111.3	0.82	24.12	24.07	24.07	0.29	−0.2	−77
$\sqrt{21}/\sqrt{73}$	4.56	5.1	111.3	0.82	20.89	20.88	20.87	0.48	−0.1	−78
$\sqrt{7}/5$	4.61	19.1	112.2	0.80	12.06	12.22	12.20	1.8	1.1	−72
$\sqrt{13}/4\sqrt{3}$	4.68	16.1	112.2	0.80	16.43	16.93	16.89	3.6	2.8	−57

Each configuration is built by combining different supercells in individual layers with a relative rotational angle *α* between them. *L*_Sn/G_ is the lattice constant of the relaxed bilayer heterostructure and the corresponding *a* is the effective lattice constant of stanene in the relaxed heterostructure, while *L*_Sn_ and *L*_G_ are the lattice constants for the particular unrelaxed supercells of stanene and graphene, respectively. *θ* and Δ are the bond angle and buckling height in stanene, respectively. The mismatch, strain, and *E*_*b*_ are defined in the text

**Fig. 1:**
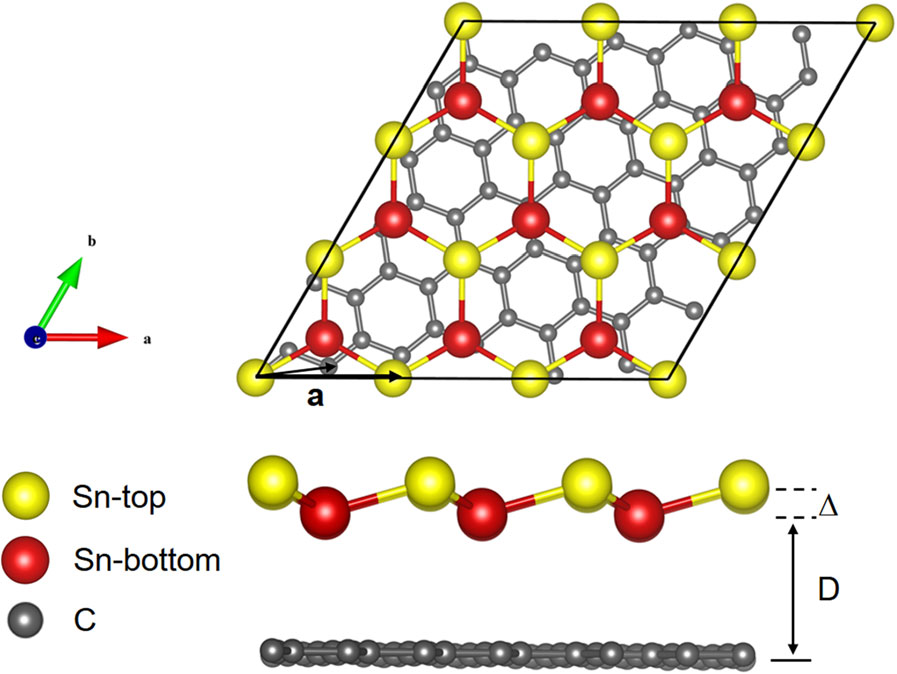
Top and side views of the heterostructure of $\mathrm{S}\mathrm{n}(3)/\mathrm{G}\left(\sqrt{31}\right)$ (**a** heterostructure consisting of 3 × 3 stanene unit cell and $\sqrt{31}\times \sqrt{31}$ graphene unit cell). *Yellow*, *red*, and *gray spheres* represent Sn-top, Sn-bottom, and C atoms, respectively. Δ and *D* represent the buckling height of stanene and interlayer distance between the substrate graphene and bottom Sn layer, respectively. The *black arrows* represent the lattice vector ${\overrightarrow{a}}_{\mathrm{Sn}}\;\mathrm{and}\;{\overrightarrow{a}}_{\mathrm{G}}$ and of the unit cell of stanene and graphene

Figure 1 shows the atomic structure of the Sn/G bilayer heterostructure system. The yellow and red atoms represent different types of Sn atoms in the low-buckled monolayer. A Sn atom in the top layer of stanene is placed on the top of a C atom in graphene. After relaxing, the buckling height Δ is found to be 0.8 Å in this system, and the interlayer distance from graphene to the bottom Sn layer is 3.5 Å based on the LDA calculation (to be discussed below), indicating that it belongs to the class of vdW type of heterostructures.

*L*_Sn/G_ in Table 1 is the heterostructure length of the fully relaxed Sn/G bilayer determined by the LDA, while *L*_Sn_ and *L*_G_ are the lattice constants for the particular unrelaxed supercells of stanene and graphene, respectively. The lattice parameter of Sn/G heterostructure is fixed to be (*L*_Sn_ + *L*_G_)/2 with a small lattice mismatch for both stanene and graphene. After fully relaxation, it is found that *L*_Sn/G_ is very close to *L*_G_, which indicates that there is almost no strain in graphene layer. The lattice mismatch between the periodic unrelaxed supercell of monolayer stanene and graphene is defined as mismatch *=* |*L*_Sn_ − *L*_G_|*/L*_G_, and it is a quite small value as shown in Table 1. The strain in the stanene layer is defined by

1 $$ \mathrm{strain} = \frac{a-{a}_0}{a_0}=\frac{L_{\mathrm{Sn}/\mathrm{G}}-{L}_{\mathrm{Sn}}}{L_{\mathrm{Sn}}}, $$

where *a* and *a*_0_ are the relaxed (bilayer) and unrelaxed primitive lattice constants.

**Fig. 2:**
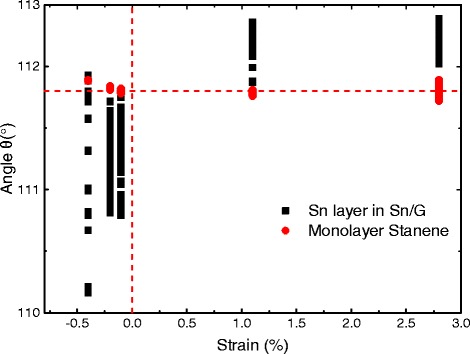
Distribution of the bond angles in Sn layer of different heterostructure models in Table 1 and corresponding monolayer stanene

As shown in Table 1, we focus on the heterostructure models that induce a strain of less than 3%. The bond angles *θ* and buckling height Δ in stanene will be slightly affected by the strain as shown in Table 1. In free-standing monolayer stanene, the bond angle *θ* is uniform, as shown as red circle in Fig. 2. With the presence of a substrate, the bond angles exhibit a small variation of a few degrees, as the lattice symmetry is slight broken in the Sn layer. *θ* shown in Table 1 is the average value, and all the distributions of bond angles are shown in Fig. 2. Obviously, when the strain < 0, the stanene layer is applied by a compressive strain, which causes the bond angle *θ* to be slightly smaller and the buckling height Δ to be higher. There are opposite results when the strain > 0, the stanene layer is forced by a tensile strain. The change of electronic properties caused by the variation of bond angles will be discussed below.

**Fig. 3:**
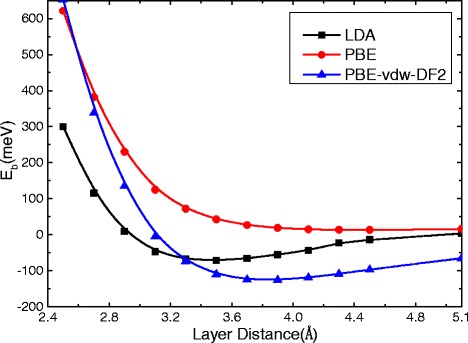
Interlayer binding energy per Sn atom of the bilayer $\mathrm{S}\mathrm{n}\left(\sqrt{7}\right)/\mathrm{G}(5)$ as a function of interlayer spacing. Results using different exchange-correlation functionals are shown. See text for the geometry and the binding energy definition

**Fig. 4:**
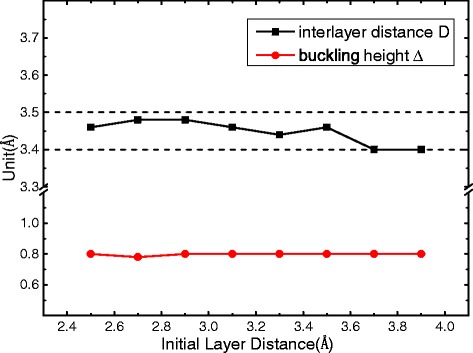
Equilibrium interlayer distance *D* of the bilayer Sn(7)/G(5) and buckling height Δ of Sn layer obtained from optimized structure of different initial interlayer distance

**Fig. 5:**
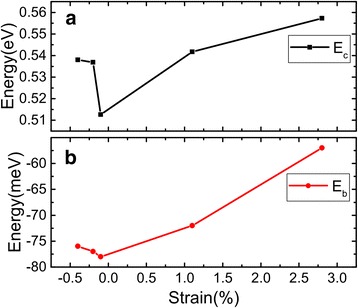
Binding energy (per Sn atom) *E*_b_ and energy per Sn in comparison with the bulk value *E*_c_ for stanene on graphene obtained using various supercell models in Table 1. The results (**a** *E*_c_ and **b** *E*_b_) are plotted as a function of strain in the layer

The vdW interaction between the layers requires special attention. To quantitatively characterize the interlayer interaction strength, we define a binding energy (*E*_b_, per Sn atom) in the Sn/G bilayer as

2 $$ {E}_{\mathrm{b}}=\frac{E_{\mathrm{Sn}/\mathrm{G}}-{E}_{\mathrm{Sn}}-{E}_{\mathrm{G}}}{N_{\mathrm{Sn}}}, $$

where *E*_Sn/G_, *E*_Sn_, and *E*_G_ represent the total energies of the Sn/G heterostructure, corresponding monolayer stanene, and monolayer graphene, respectively, and *N*_Sn_ is the number of Sn atoms in this structure. This binding energy for the $\mathrm{S}\mathrm{n}\left(\sqrt{7}\right)/\mathrm{G}(5)$ bilayer is evaluated by various exchange-correlation functionals, and the results as a function of the layer separation are shown in Fig. 3. Except for the PBE-GGA that fails to create any binding between the layers, other functionals (LDA and PBE-vdW-DF2) predict energy minima at an interlayer separation around 3.5–3.7 Å. The LDA gives an energy lowering of 72 meV per Sn atom due to the interlayer interaction, which is higher about 70 meV than the case of explicit PBE vdW calculations, suggesting that the results of PBE vdW method are more credible due to the interlayer interaction. Since we are mostly concerned with relative energies and the electronic structure in the present work, and the variation in the interlayer separation around 3.5–3.7 Å is not expected to significantly affect the results. In the following, we will report LDA results at an interlayer separation of 3.5 Å for the simplicity of the calculations. As shown in Table 1 and *E*_b_ in Fig. 5b, the smallest strain structure $\mathrm{S}\mathrm{n}\left(\sqrt{21}\right)/\mathrm{G}\left(\sqrt{73}\right)$ has the lowest binding energy, which is −78 meV per Sn atom, corresponding the strongest binding effect. The value is similar to that of graphene/silicene (−66 meV per C atom) within a plane-wave basis set [40]. It indicates that our calculations for *E*_b_ were reliable. The weak vdW interactions dominate between stanene and graphene, suggesting that graphene can be used as an ideal substrate for stanene. Moreover, to verify the reliability of the equilibrium interlayer distance *D*, different initial layer distance (2.5–3.9 Å) between stanene and graphene has been fully relaxed, getting up to an identical distance (3.4–3.5 Å) and a uniform buckling height Δ = 0.8 Å as shown in Fig. 4.

The energetics of the stanene overlayer can be addressed by examining the energy per Sn atom defined as:

3 $$ {E}_{\mathrm{c}}=\frac{E_{\mathrm{Sn}/\mathrm{G}}-{E}_{\mathrm{G}}}{N_{\mathrm{Sn}}}-{\mu}_{\mathrm{Sn}}, $$

where *μ*_Sn_ is the chemical potential set to the energy per atom of bulk Sn. The calculated energies per Sn atom using different heterostructures in Table 1 are plotted as a function of the strain in Fig. 5a. The value of *E*_c_ is positive, indicating that the 2D structure is higher in energy than the 3D diamond structure. Among all the heterostructures we have considered, $\mathrm{S}\mathrm{n}\left(\sqrt{21}\right)/\mathrm{G}\left(\sqrt{73}\right)$ has the smallest strain (−0.1%) and the lowest energy as expected. The energy difference per atom between different supercell models is smaller than the thermal energy at room temperature (about 26 meV), indicating that multiple phases of different crystalline orientation could coexist at room temperature.

### Electronic Structure

**Fig. 6:**
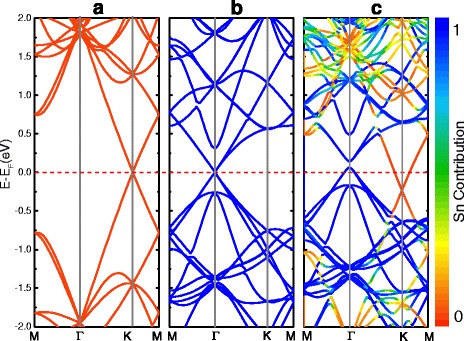
Electronic structures of the **a** monolayer graphene, **b** monolayer stanene, and **c** bilayer $\mathrm{S}\mathrm{n}(3)/\mathrm{G}\left(\sqrt{31}\right)$. The *red line* represents the Fermi level, which set to be zero. The relative contribution of stanene is coded by color: *blue* (*red*) corresponds to the state originating only from stanene (graphene). The substrate-induced gap is 67 meV for Sn (Γ) and 3 meV for graphene (K)

Two-dimensional honeycomb structures exhibit a symmetry between the sublattices and therefore have a linear energy dispersion in the vicinity of the Dirac points at K point. Figure 6c shows the projected band structures of $\mathrm{S}\mathrm{n}(3)/\mathrm{G}\left(\sqrt{31}\right)$. The relative contribution of stanene is coded by color, in which blue (red) corresponds to the state originating only from stanene (graphene). For comparison, the energy band structures of the corresponding graphene and isolated stanene monolayer are also show in Fig. 6a, b. For $\mathrm{S}\mathrm{n}(3)/\mathrm{G}\left(\sqrt{31}\right)$, the electronic structure is not a simple sum of those of each constituent. Forcing on the position of Dirac point reference to the Fermi level, we found a significant change. The Dirac point of stanene locates at Γ shift 0.1 eV above the Fermi level, while graphene moves 0.2 eV below the Fermi level. Meanwhile, it can be seen that, at the Γ point, the π and π* bands repulse each other, forming a band gap as large as 67 meV. The Fermi level crosses the two Dirac zones of stanene and graphene, inducing weak p-type and n-type doping of them, respectively, and generating a small amount of electron transfer from stanene to graphene. Based on the linear Dirac-like dispersion relation *E*(*k*) = ± *ℏν*_*F*_|*k*| around the Fermi levels [55], the charge carrier (hole or electron) concentration of doped graphene can be estimated by the following equation [56, 57]:

4 $$ {N}_{h/e}=\frac{{\left(\varDelta {E}_{\mathrm{D}}\right)}^2}{\ \pi \Big(\hslash {\nu_{\mathrm{F}\Big)}}^2}, $$

where *ΔE*_D_ is the shift of graphene’s Dirac point (*E*_D_) relative to the Fermi level (*E*_F_), that is *ΔE*_D_ = *E*_D_ − *E*_F_. Our calculated charge carrier concentrations are *N*_h_ (Sn) = 1.4 × 10^12^ cm^−2^ and *N*_e_ (G) = 1.6 × 10^11^ cm^−2^ for stanene and graphene in bilayer, respectively. These values are larger than the intrinsic charge carrier concentration of graphene at room temperature ( *n* = πk_B_^2^T^2^/6*ℏ*ν_F_^2^ = 6 × 10^10^cm^− 2^) [58]. Furthermore, the charge carrier concentrations of both stanene and graphene in Sn/G heterostructure can be tuned via the interfacial spacing [59]. The self-doping phenomenon in Sn/G heterostructure provides an effective and tunable way for new optoelectronic devices.

**Fig. 7:**
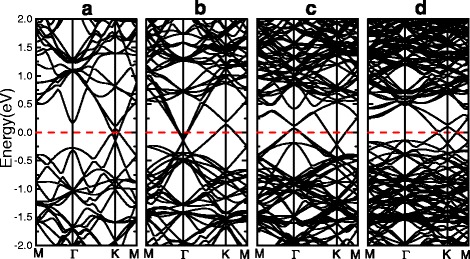
Band structure of **a** $\mathrm{S}\mathrm{n}\left(\sqrt{7}\right)/\mathrm{G}(5)$, the Dirac point of them both located at K, **b** $\mathrm{S}\mathrm{n}\left(\sqrt{13}\right)/\mathrm{G}\left(4\sqrt{3}\right)$, the Dirac point of stanene (graphene) is located at K (Γ), **c** $\mathrm{S}\mathrm{n}\left(\sqrt{21}\right)/\mathrm{G}\left(\sqrt{73}\right)$, the Dirac point of stanene (graphene) is located at Γ (K), and **d** $\mathrm{S}\mathrm{n}\left(2\sqrt{7}\right)/\mathrm{G}\left(\sqrt{97}\right)$, the Dirac points of stanene and graphene are both located at K

Figure 7 shows the band structure of other four supercell models. We can find that the position of the Dirac point located at different high-symmetry point results from the band-folding caused by the various supercell. The graphene substrate introduces an inhomogeneous potential that breaks the sublattice symmetry of stanene. For a free-standing stanene monolayer, the bond angles are uniform. For the graphene-supported layer, the bond angles have a variation, as shown in Fig. 2; hence, the sublattice symmetry is broken, and a gap is opened. And the opened gaps at the Dirac point for $\mathrm{S}\mathrm{n}\left(\sqrt{7}\right)/\mathrm{G}(5)$, $\mathrm{S}\mathrm{n}(3)/\mathrm{G}\left(\sqrt{31}\right)$, $\mathrm{S}\mathrm{n}\left(\sqrt{13}\right)/\mathrm{G}\left(4\sqrt{3}\right)$, $\mathrm{S}\mathrm{n}\left(\sqrt{21}\right)/\mathrm{G}\left(\sqrt{73}\right)$, and $\mathrm{S}\mathrm{n}\left(2\sqrt{7}\right)/\mathrm{G}\left(\sqrt{97}\right)$ are 34, 67, 53, 44, and 22 meV, respectively. The spin–orbit coupling effect is also calculated, and the gap at the Dirac point will reduce after adding the SOC. The results suggest that the gap in the bilayer heterostructure could be tuned by the interplay between the substrate and SOC effects, moreover by voltage or strain. The characteristics of the Dirac fermions are preserved as shown in Fig. 7b, c accompanied with a small amount of charge transfer from stanene to graphene, as discussed in $\mathrm{S}\mathrm{n}(3)/\mathrm{G}\left(\sqrt{31}\right)$.

**Fig. 8:**
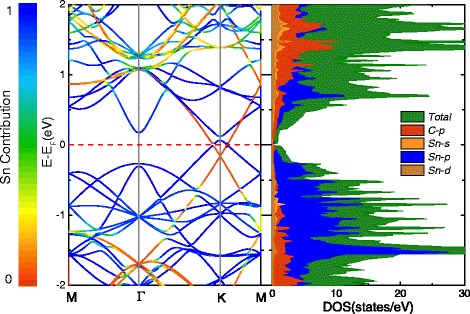
Electronic band structure and DOS of $\mathrm{S}\mathrm{n}\left(\sqrt{7}\right)/\mathrm{G}(5)$. The relative contribution of stanene is coded by color: *blue* (*red*) corresponds to the state originating only from stanene (graphene)

When focusing on Fig. 7a, d, we find that the Dirac points of stanene and graphene are located at the same high-symmetry point K. To investigate the mechanisms of action more clearly, the projected band structure and density of states (DOS) of $\mathrm{S}\mathrm{n}\left(\sqrt{7}\right)/\mathrm{G}(5)$ is shown in Fig. 8. It is clearly seen that there exists a band inversion around the Fermi level at K point. For stanene, the original valence band shifts up to the conduction band, while for graphene, the original conduction band turns into valence band which is below the Fermi level. Meanwhile, both the maximum valence band and minimum conduction band are transformed from the “cone shape” to the “Mexican-hat shape,” leading to the appearance of two Dirac feature points with the band gap about 34 meV around the K point. The band inversion associated with the change of band shapes is reminiscent of many topological insulators (TIs) [60, 61]. In order to ascertain the topological phase transition in the $\mathrm{S}\mathrm{n}\left(\sqrt{7}\right)/\mathrm{G}(5)$ heterostructure, we calculate the Z2 topological invariants. We implement the method proposed by Soluyanov and Vanderbilt [62], in which the 2D Z2 invariant is obtained by counting the number of jumps of the “biggest gap” among the 1D hybrid Wannier charge centers [63] (WCCs) during the evolution. Although the results have shown that it exhibits a topologically trivial phase, it would provide a way and useful guideline for the investigation the QSH insulator and the grown of stanene or other 2D vdW heterostructures.

## Conclusions

In conclusion, by first-principle calculations, we found it is possible to synthesize stanene on the graphene substrate without destroying its characteristics of the Dirac-fermion-like linear dispersion around Dirac points, due to the weak van der Waals interlayer interaction. In addition, multiple phases of different crystalline orientation of stanene and graphene could coexist at room temperature based on our energetics analysis. Moreover, interlayer interactions in stanene and graphene heterostructure can induce tunable band gaps at stanene’s Dirac point, and weak p-type and n-type doping of stanene and graphene, respectively, generating a small amount of electron transfer from stanene to graphene. For stanene on graphene, the gap created by the substrate effect is of the same order as that induced by the SOC effect. Interestingly, for model $\mathrm{S}\mathrm{n}\left(\sqrt{7}\right)/\mathrm{G}(5)$, there exists a band inversion around the Dirac zones at K point and emerges a band gap about 34 meV overall the band structure, indicating that it shows a semiconductor feature. Our fundamental study of the structural and electronic properties of these stanene/graphene heterostructures may provide important insight and useful guideline for the grown and applications of stanene or other 2D vdW heterostructures.
